# Optimization of preparation conditions and performance of a new degradable soil water retaining agent

**DOI:** 10.1038/s41598-024-60784-2

**Published:** 2024-05-19

**Authors:** Zhang Yumang, Wang Yongheng, Wang Chengyu, Gao Yunhang, Liu Shuxia, Xie Zhonglei, Chang Hongyan

**Affiliations:** 1 College of Resources and Environment, Jilin Agricultural University, Changchun, 130118 China; 2 Key Laboratory of Sustainable Utilization of Soil Resources in Commodity Grain Base of Jilin Province, Changchun, 130118 China; 3 College of Life Sciences, Changchun University of Science and Technology, Changchun, 130600 China; 4 College of Plant Science, Jilin University, Changchun, 130062 China; 5 College of Animal Science and Technology, Jilin Agricultural University, Changchun, 130118 China

**Keywords:** Degradable soil water retaining agent, High water absorption, Polyaspartic acid, Bentonite, Preparation and performance, Agroecology, Plant sciences, Plant ecology

## Abstract

Using polyaspartic acid (PAsp) and bentonite (BT) as the main raw materials, a new type of degradable soil water retaining agent (PAsp-AA/BT) was synthesized by microwave radiation. The optimum synthesis conditions and comprehensive properties of PAsp-AA/BT were discussed and the structure and surface characteristics of PAspsp-AA/BT were characterized by FTIR, SEM, XRD and TGA in the paper. The results showed that the optimum synthesis conditions of PAsp-AA/BT were as follows: the dosages of polyaspartic acid (PAsp), bentonite (BT), initiator potassium persulfate, crosslinking agent *N*,*N*′-methylene bisacrylamide was 5, 3, 0.3, 0.03%, respectively, the neutralization degree of acrylic acid was 75%, and the microwave power was 490W. Under this condition, the absorption ratio of the synthesized PAspsp-AA/BT in deionized water and 0.9% NaCl solution was 953 and 164 g/g, respectively. The synthesized PAsp-AA/BT had a high water absorption rate, good water retention and repeated water absorption, and the degradation rate in soil within 30 days reached 32.75%, with good degradation effect. The analysis of SEM, FT-IR, XRD and TGA showed that: the surface of PAsp-AA/BT was rough and had obvious pore structure, which was conducive to the diffusion of water molecules; polyaspartic acid, bentonite and acrylic acid were polymerized; the cross-linking structure was formed between polyaspartic acid, bentonite and acrylic acid; the product of PASP-AA/BT had good thermal stability. This study provides a new soil water retaining agent, which is helpful for the better development of soil water retaining agent research.

## Introduction

Water, an important component of soil ecosystems, is essential for nutrient cycling and crop productivity^1–4^. Recently, global climate change has accelerated the frequency and intensity of drought events, as well as their potential consequences^5^. Due to the influence of geographical location and climate conditions, the area of water shortage regions in the world is increasing^6–10^. In 2023, drought affected 6090.2 thousand hectares of crops in China, resulting in a direct economic loss of 51.28 billion. At the same time, with the accelerating process of industrialization and urbanization, industrial water and domestic water will rise significantly^11,12^. Water retaining agent, as a superabsorbent polymer (SAPs), is a polymer material with a three-dimensional network structure, containing hydroxyl, carboxyl, amide and other strong hydrophilic groups, and has a certain crosslinking effect^7^. The water-retaining agent has good adsorption performance and can absorb the water in the environment and use it reciprocately. However, most water retaining agents are harmful to the soil environment because of their own, such as polypropylene and other degradation difficulties. Accompanied by environmental hazards, denatured starch and other water absorption is low and the application amount is too large.

Actually, the versatile characteristics of non-toxicity and high water adsorption rate of PAsp hydrogel indeed lead to its wide applications as a water-retaining agent for the ecological restoration in the desert area. Wei et al.^8^. reported that adding chemicals can also play a role in water retention, but considering the environment, plants and other factors, polyaspartic acid has the characteristics of degradation, does not affect the environment. PAsp based composite superabsorbent a biodegradable material with high water absorption. The large number of carboxyl groups in PAsp can bind water on the PAsp side chain, while the gel structure of PAsp can spatially absorb water from the environment^9^. Due to the simple composition and structure, PAsp based hydroscopic resins suffer from disadvantages such as poor water retention, salt resistance and thermal stability, which limit their applications in many fields^10,13^.

Bentonite (BT) is a non-metallic mineral with montmorillonite as the main mineral component^14^. The montmorillonite structure is a 2:1 type crystal structure consisting of two silicon oxide tetrahedrons sandwiched with a layer of aluminum oxide octahedron. BT can be used as a soil conditioner, which has good effect on retaining soil water and nutrients. Some studies have shown that the application of BT can improve the water retention performance of sandy soil. At the same time, there are a large number of Cu^2+^, Mg^2+^, and other cations with poor stability, which are prone to ion exchange^15,16^. BT has strong hygroscopicity and expansibility, and can absorb 8–15 times of its own volume of water, and its volume expansion can reach several to 30 times^17^. BT can form a dispersion suspension gel with certain viscosity and lubricity in the medium^17^. BT has good cation exchange capacity, and the liquid adsorption rate can reach 5 times its own weight^14,18^.

In this study, PAsp and BT were used to prepare degradable soil water retaining agent and characterize its performance, the main purposes follows: (1) using PAsp and BT as raw materials, partially neutralized AA as monomer, KPS as initiator, and MBA as cross-linking agent, biodegradable soil water retaining agent was prepared by microwave polymerization, meanwhile, single factor variable test was used to explore the effects of different raw materials, amount of crosslinking agent and initiator, neutralization degree, microwave power and microwave time on the liquid absorption ratio of water retaining agent, and determined the optimal synthesis conditions^19–23^; (2) the properties of degradable soil water retaining agent prepared under the optimal synthesis conditions, such as liquid absorption rate, water retention rate, degradation rate and repeated water absorption, were discussed^24,25^; (3) the structure of the degradable soil water retaining agent was characterized by FT-IR, XRD, SEM and TGA. The study will provide new methods and materials for land desertification control and drought resistance and water conservation in agriculture and forestry^26–31^.

The change of preparation conditions of water retaining agent has a great impact on the performance of water retaining agent. In this study, PAsp and BT were used as the main raw materials, and a new degradable water retaining agent was obtained by microwave radiation method. Compared with the current water retaining agent sold in the market, the water absorption ratio of water retaining agent was improved, and the decomposition rate of water retaining agent was increased. It retains water and has no adverse effects on the soil.

## Materials and methods

### Materials

Polyaspartic acid (PAsp, industrial grade) was obtained from Shijiazhuang Desai Chemical Co., Ltd., China,Light yellow powder,pH 9.0,Molecular weight: 133.102; Bentonite (BT, chemically pure) was from SOLEBAR Biotechnology Co., Ltd., Beijing, China,Hardness 2, density 2.5 g/cm^3^, 250-mesh screen; Acrylic acid (AA, analytical grade) was from Shanghai Yien Chemical Technology Co., Ltd., China,molecular weight is 72.06, Density 1.051 g/cm^3^; Potassium persulfate (KPS, analytical grade) was from Tianjin Yongsheng Fine Chemical Co., Ltd., China,Molecular weight 270.322, density 2.47 g/cm^3^; *N*,*N*′- methylenebisacrylamide (MBA, analytical grade) was from Aladdin Reagent Co., Ltd,Molecular weight: 154.17, relative density: 1.352, melting point: 184 °C. All reagents were analytical grade, and all solutions were prepared with distilled water.

### Preparation method of water retaining agent

The synthesis method of degradable soil water retaining agent was as follows: accurately measured 5 mL of AA in a beaker, slowly added 20% NaOH solution in an ice water bath to neutralize it to a certain degree of neutralization, cooled it to room temperature, then added a certain amount of BT and PAsp in turn, stirred it evenly, then added a certain amount of KPS and MBA , stirred it evenly again, quantified with deionized water. Used an ultrasonic cleaner to disperse the mixed solution evenly and then transferred it to a microwave oven. Reaction take placed under a certain radiation power. After the reaction completed, took out the product. After cooling, added methanol solution to soak for 12 h to remove residual monomers on the surface. Finally, put the product into a 75 °C drying oven to dry for 24 h. Crushed and sieved to obtain a water retaining agent sample.

### Determination method of properties of water retaining agent

1. Determination of liquid absorption ratio.

Accurately weighed a certain amount of dried water retaining agent sample into a 200 mesh tea bag, respectively put a certain amount of deionized water and 0.9% NaCl solution into the bag, made it fully swollen, hanged it for standing until there was no liquid drop, and accurately weighed the gel mass after swelling balance. Calculated the liquid absorption ratio Q of the water retaining agent according to the following formula:

$$\begin{array}{*{20}c} {{\text{Qd(Qs)}} = \frac{{{\text{m}}_{{2}} - {\text{m}}_{{1}} }}{{{\text{m}}_{{1}} }}} \\ \end{array}$$

where Qd—ratio of water absorbed and deionized, g/g; Qs—0.9% NaCl solution absorption ratio, g/g; M_1_—mass of dry water retaining agent sample, g; M_2_—gel mass in swelling equilibrium, g.

(2) Determination of liquid absorption rate.

Accurately weighed a certain amount of dry water retaining agent sample into a 200 mesh tea bag, put a certain amount of deionized water into it to make it fully swollen, took it out every other period of time, hanged it until there was no liquid drop, and accurately weighed the quality of gel after swelling and balancing at different times. Calculated the liquid absorption ratio Q of the water retaining agent according to the following formula, and studied the liquid absorption rate by measuring the change rule of the liquid absorption ratio with time.

$$\begin{array}{*{20}c} {{\text{Qt}} = \frac{{{\text{m}}_{{4}} - {\text{m}}_{{3}} }}{{{\text{m}}_{{3}} }}} \\ \end{array}$$

where Qt—liquid absorption ratio at different times, g/g; M_3_—mass of dry water retaining agent sample, g; M_4_—gel mass at different time of swelling equilibrium, g.

(3) Determination of water retention rate.

Accurately weighed a certain amount of swelling balanced gel, put it into a 60 °C constant temperature blast drying oven, weighed it at a certain interval, and measure the quality of gel at different times. Calculated the water retention ratio of the water retaining agent as follows:

$$\begin{array}{*{20}c} {{\text{t}} = \frac{{{\text{m}}_{{5}} }}{{{\text{ m}}_{{6}} }}} \\ \end{array}$$

where φt—water retention rate at time t, %; M_5_—gel mass at time t, g; M_6_—swelling equilibrium gel mass, g.

(4) Determination of degradation rate.

Accurately weighed a certain amount of dry water retaining agent sample into a 200 mesh tea bag, and buried it in a culture box filled with soil, with a soil depth of 20 cm. Took out the samples after burial for 5, 10, 20 and 30 d respectively, removed the topsoil on the surface of the tea bag with a brush, dried them in a drying oven to constant weight, and observed the surface morphology of the water retaining agent samples taken at different times with a scanning electron microscope. Calculated the degradation rate of the water retaining agent according to the following formula α:

$$\begin{array}{*{20}c} {{\alpha t} = \frac{{{\text{m8}} - {\text{m7}}}}{{{\text{ m}}_{{8}} }}} \\ \end{array}$$

where α t—degradation rate at different time, %; M7—mass of dry water retaining agent sample at different times, g; M8—mass of dry water retaining agent sample, g.

### Structural characterization of water retaining agent

1. FT-IR analysis
  Mixed the sample powder of dry water retaining agent with potassium bromide powder, grinded it in agate mortar, and then pressed it. The scanning range was 500–4000 cm^−1^, and the resolution of the instrument was 2 cm^−1^.
2. Scanning electron microscope (SEM) analysis
  The sample powder of the dry water retaining agent was sprayed with gold, and its surface morphology was observed by scanning electron microscope.
3. X-ray diffraction (XRD) analysis
  Put the dry water retaining agent sample into an agate mortar and grinded it to a particle size of about 2 μm. It was then placed in a grooved glass sheet and scanned continuously with an X-ray diffractometer. The experimental conditions were CuK µ radiation, voltage 40 kV, scanning range 2–80°, scanning speed 2°/min.
4. Thermogravimetric analysis (TGA)
  Put the sample powder of the dry water retaining agent into the thermogravimetric analyzer for thermogravimetric analysis. The experimental conditions were N_2_ purging, the temperature range was 20–750 °C, and the heating rate was 10 °C /min.

## Results and discussion

### Optimization of preparation conditions of degradable soil water retaining agent

1. Effect of PAsp amount on absorbing ratio of water retaining agent.

**Figure 1:**
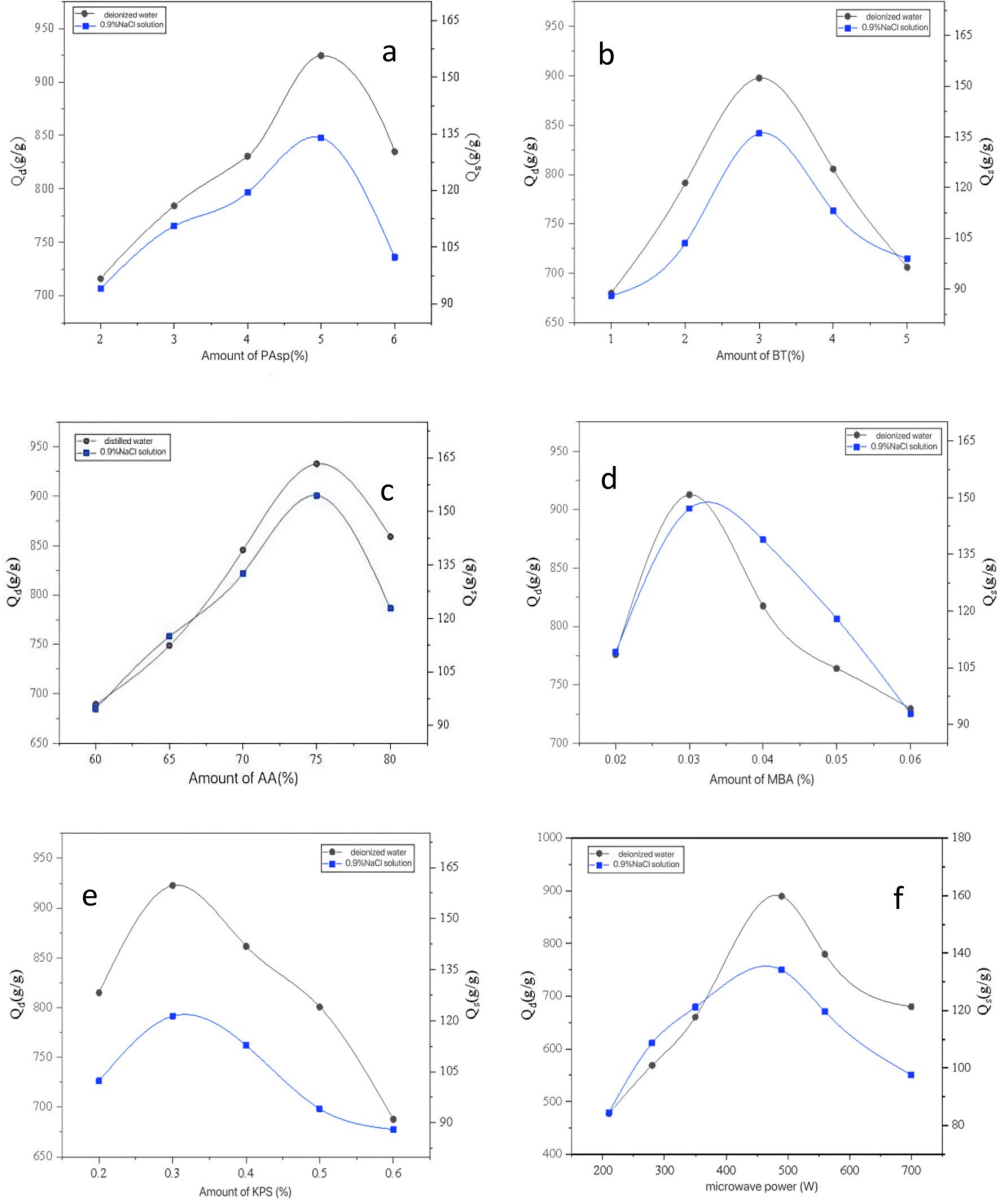
Effect of reactant application on properties of water retaining agent.

It can be seen from Fig. 1a that with the continuous increase of the amount of PAsp, the liquid absorption ratio of the water retaining agent in deionized water and 0.9% NaCl solution increased first and then decreased, the maximum was 924 and 134 g/g, respectively, when the amount of PAsp was 5% of the monomer mass.

With the increase of PAsp content, the cation content and polymer branch chain of the water retaining agent were increased, the osmotic pressure of the water retaining agent was increased, and the water retaining performance of the water retaining agent was improved. Especially, the increase of the amount of PAsp improved obviously the absorption ratio of water retaining agent in 0.9% NaCl solution. When the dosage of PAsp was further increased, the proportion of carboxyl, amino and other hydrophilic groups in the water retaining agent was reduced, the cross-linking concentration was too large, and the network structure was too dense, thus reducing the liquid absorption ratio. Wutang Sang et al. showed that with the addition of PAsp, the water retention performance of the water retaining agent showed a trend of first increasing and then decreasing, and the maximum water retention rate was reached when the PA level reached 20%^31^.

(2) Effect of BT dosage on liquid absorption ratio of water retaining agent.

Figure 1b shows that the change trend of the liquid absorption ratio of the water retaining agent in deionized water and 0.9% NaCl solution increased at first and decreased later with the increasing amount of BT. Niu Yuhua research showed that the addition of BT increased the specific surface area of the water retaining agent, and the fold area increased significantly^32^. When the amount of bentonite was 3% of the mass of monomer, the liquid absorption ratio reached the maximum values of 897 and 136 g/g, respectively. With the increase of BT dosage, the cross-linking point of water retaining agent increased, the cross-linking density also increased and formed a very dense polymer network, and its space volume became smaller, thus affecting the liquid absorption ratio. When the amount of BT was less, the cross-linking structure of the water retaining agent reduced, and the liquid absorption ratio also reduced accordingly.

(3) Effect of AA on absorbing ratio of water retaining agent.

Figure 1c shows that when the neutralization degree was 75%, the liquid absorption rate of the water retaining agent was the highest, and when the neutralization degree exceeded 75%, the liquid absorption rate of the water retaining agent in deionized water and 0.9% NaCl solution had a downward trend. After the acrylic acid was neutralized by NaOH solution, NaOH converted the group of –COOH into more hydrophilic –COONa. Tang research shows that with the increase of the neutralization degree of AA, the water absorption capacity will begin to decrease significantly when the saturation exceeds 65%, because AA carries Na ions, increases the osmotic pressure, and thus improves the absorption force^33^. According to Flory's theory, with the increased of neutralization degree, the content of strong hydrophilic group -COONa also increased. Its dissociation changed the charge density of the water retaining agent, leading to the change of internal and external osmotic pressure, making it easier for water molecules to enter the water retaining agent, thus improving the liquid absorption ratio. However, since there were many exchangeable cations and charges on the surface and between layers of bentonite, the charge density can be adjusted. When the degree of neutralization was lower than 75%, the degree of neutralization increased and the liquid absorption ratio increased. When the degree of neutralization was higher than 75%, the liquid absorption ratio decreased due to the excessive ion concentration.

(4) Effect of crosslinking agent amount on absorbing ratio of water retaining agent.

Figure 1d shows that with the increase of the amount of crosslinking agent, the liquid absorption ratio of the water retaining agent in deionized water and 0.9% NaCl solution had a trend of increasing first and then decreasing, and the liquid absorption ratio reached the maximum when the amount of crosslinking agent was 0.03% of the monomer mass, similar to the results of Qiao’s study^34^. When the amount of crosslinking agent was small, the crosslinking degree of water retaining agent was too low, the network structure formed between molecules was incomplete, and there were more water-soluble products, resulting in low liquid absorption ratio. When the amount of cross-linking agent was too high, the cross-linking density was too large, the network structure formed between molecules was too dense, which reduced the pore diameter of the molecular network, resulting in a decrease in the liquid absorption ratio of the water retaining agent.

(5) Effect of initiator dosage on liquid absorption ratio of water retaining agent.

Figure 1e shows that when the amount of initiator was 0.3% of the monomer mass, the water retaining agent had the highest liquid absorption ratio, which was 922 g/g in deionized water and 121 g/g in 0.9% NaCl solution, respectively. It can be seen that with the increase of KPS concentration, the adsorption capacity of water retaining agent in water increases first and then decreases^35^. When the amount of crosslinking agent was fixed, the amount of initiator affected the molecular weight of the product, which was closely related to the degree of crosslinking. When the amount of initiator was too low, the free radicals in the reaction system were less, leading to incomplete crosslinking reaction and more soluble components in the product, and further reducing the liquid absorption ratio. When the amount of initiator was too large, there were more free radicals in the reaction system, the polymerization rate was faster, the chain termination reaction increased, the molecular weight of the product decreased, and the crosslinking density increased, thus reducing the liquid absorption ratio of the water retaining agent.

(6) Effect of microwave power on absorbing ratio of water retaining agent.

Figure 1f shows that the water retaining agent had the highest liquid absorption ratio when the microwave power was 490W, and the liquid absorption ratio in deionized water and 0.9% NaCl solution was 889 and 134 g/g, respectively. The microwave radiation power determines the reaction rate, thus affecting the reaction time. Microwave radiation can accelerate the generation of free radicals, thus speeding up the reaction process, which was conducive to the composition of water retaining agent. When the radiation power was less than 490 W, the more free radicals generated by radiation, the faster the reaction rate was, and the water absorption network structure formed more rapidly with the increase of the power. When the power exceeded 490W, with the further increase of the power, on the one hand, the coupling and disproportionation reactions of free radicals increased, which reduced the degree of crosslinking; on the other hand, excessive power destroyed the structure of the bentonite layer, which cannot form an effective crosslinking point. Moreover, the temperature raised too fast, and the water retaining agent was easy to harden, or even scorched, which makes the water absorption performance of the synthesized water retaining agent poor.

### Structural characterization of water retaining agent

1. FT-IR analysis.

**Figure 2:**
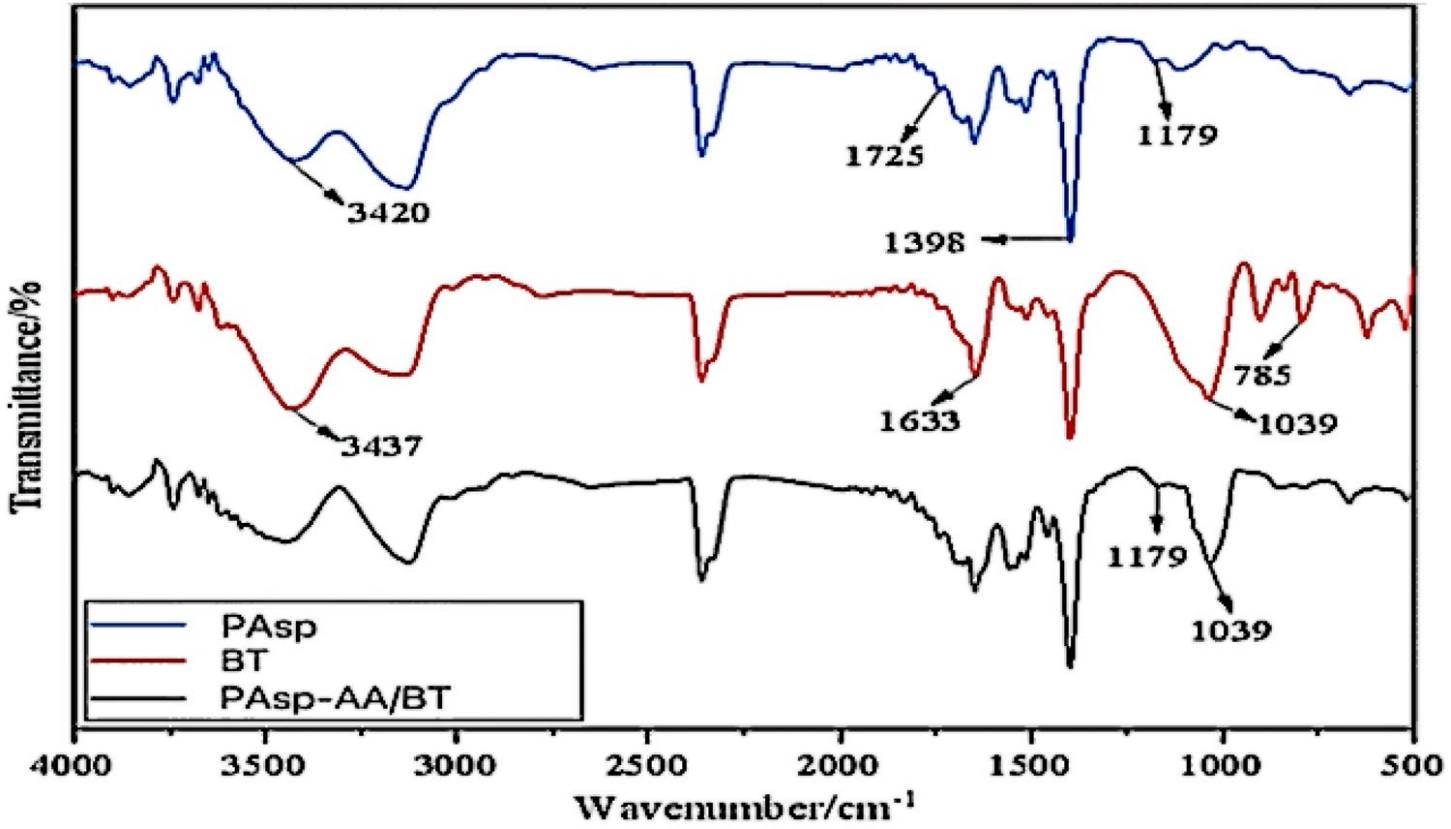
Infrared spectrum of degradable soil water-retaining agent.

Figure 2 is the infrared spectra of PAsp and BT and water retaining agent. It shows that the stretching vibration absorption peak of C–O bond in carboxyl group is near 1179 cm^−1^, the stretching vibration absorption peak of C=O in carboxyl group is near 1725 cm^−1^, the wide peak near 3420 cm^−1^ is the bending vibration of –OH, and the stretching vibration absorption peak of C–N bond in amide is near 1398 cm^−1^^36^. The infrared spectrum of BT shows that the absorption peak of Mg–Al–OH stretching vibration bond is near 785 cm^−1^, the absorption peak of Si–O–Si stretching vibration is near 1039 cm^−1^, the bending vibration absorption peak of –OH is near 1633 cm^−1^ (because there is crystal water in the lattice of bentonite), and the wide peak near 3437 cm^−1^ is the stretching vibration of interlayer adsorbed water OH. The infrared spectrum of degradable soil water retaining agent shows that the characteristic peaks of PAsp and BT appeared near 1179 and 1039 cm^−1^, respectively, indicating that both PAsp and BT participated in the polymerization reaction.

(2) X-ray diffraction (XRD) analysis.

**Figure 3:**
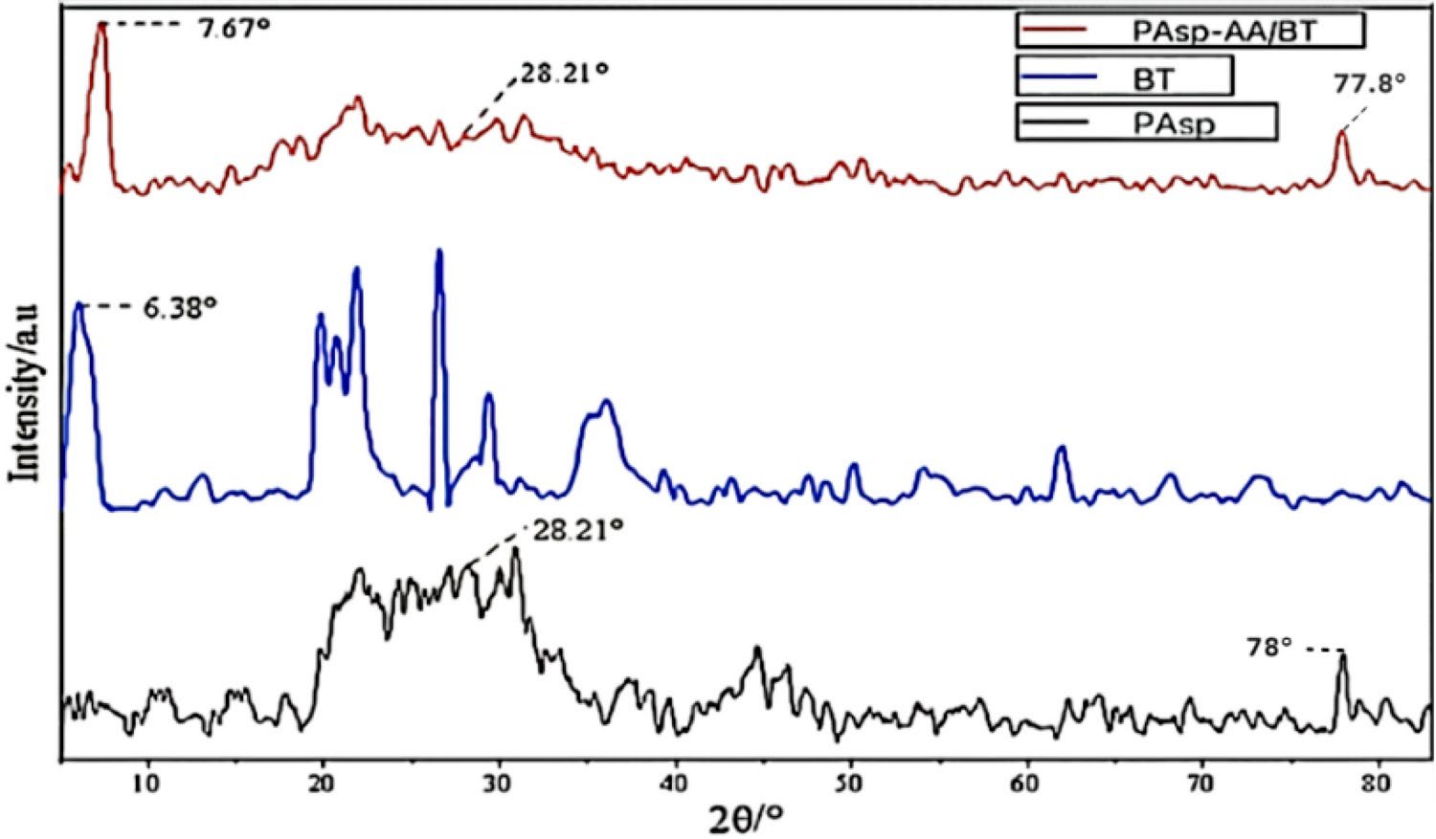
X-ray diffraction pattern of degradable soil water-retaining agent.

Figure 3 is the X-ray diffraction patterns of PAsp, BT and degradable soil water retaining agent. It can be seen from the XRD pattern of BT that there is a relatively wide diffraction peak at 2θ = 6.38°, but there is no such wide peak in the PAsp pattern. Therefore, it can be determined that this is the characteristic diffraction peak of BT. Similarly, in the XRD pattern of PAsp, a diffraction peak that does not exist in BT appears at 2θ = 28.21°and 2θ = 78°, so it can be determined that this is the characteristic diffraction peak of PAsp. According to the XRD pattern of degradable soil water retaining agent, the characteristic diffraction peak of BT appeared at 2θ = 7.67°, and the peak position shifted, and the peak intensity also weakened. The layered structure of montmorillonite, the main component of BT, was stripped and its order decreased, indicating that BT was involved in the synthesis of degradable soil water retaining agent. Similarly, the characteristic diffraction peak of PAsp appeared at 2θ = 28.21° and 2θ = 77.8°, its intensity was also weakened, indicating that PAsp also participated in the synthesis.

(3) Scanning electron microscope (SEM) analysis.

**Figure 4:**
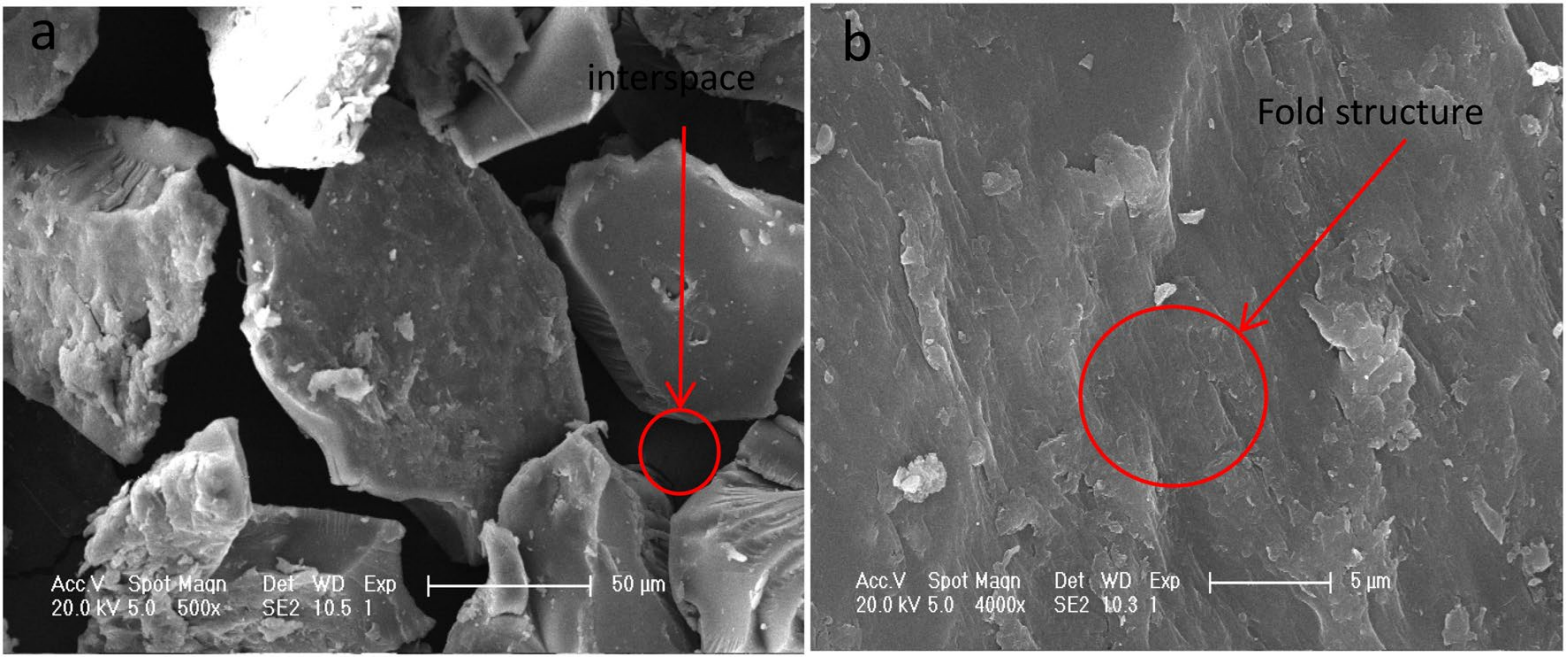
SEM of degradable soil water-retaining agent. *Note* (**a**)SEM image of degradable soil water retaining agent magnified 500 times; (**b**)SEM image of degradable soil water retaining agent magnified 4000 times.

Figure 4 shows the scanning electron microscopy of the degradable water-retaining agent magnified 500 times (a) and 4000 times (b). In Fig. 4a, it can be found that the water-retaining agent shows roughness and has an obvious void structure, which increases the specific surface area and thus increases the contact between water and water-retaining agent. In Fig. 4b, magnified 4000 times, found that after the introduction of BT, the surface of the structure became rough and loose, and many creases could be observed, which indicated that moisture was more suitable in the polymer network, which was conducive to water absorption. This result also explained that BT was directly filled into the PAsp-AA/BT structure and uniformly dispersed in the matrix without aggregation, which had a good effect on the formation of high water absorption of water retaining agent.

**Figure 5:**
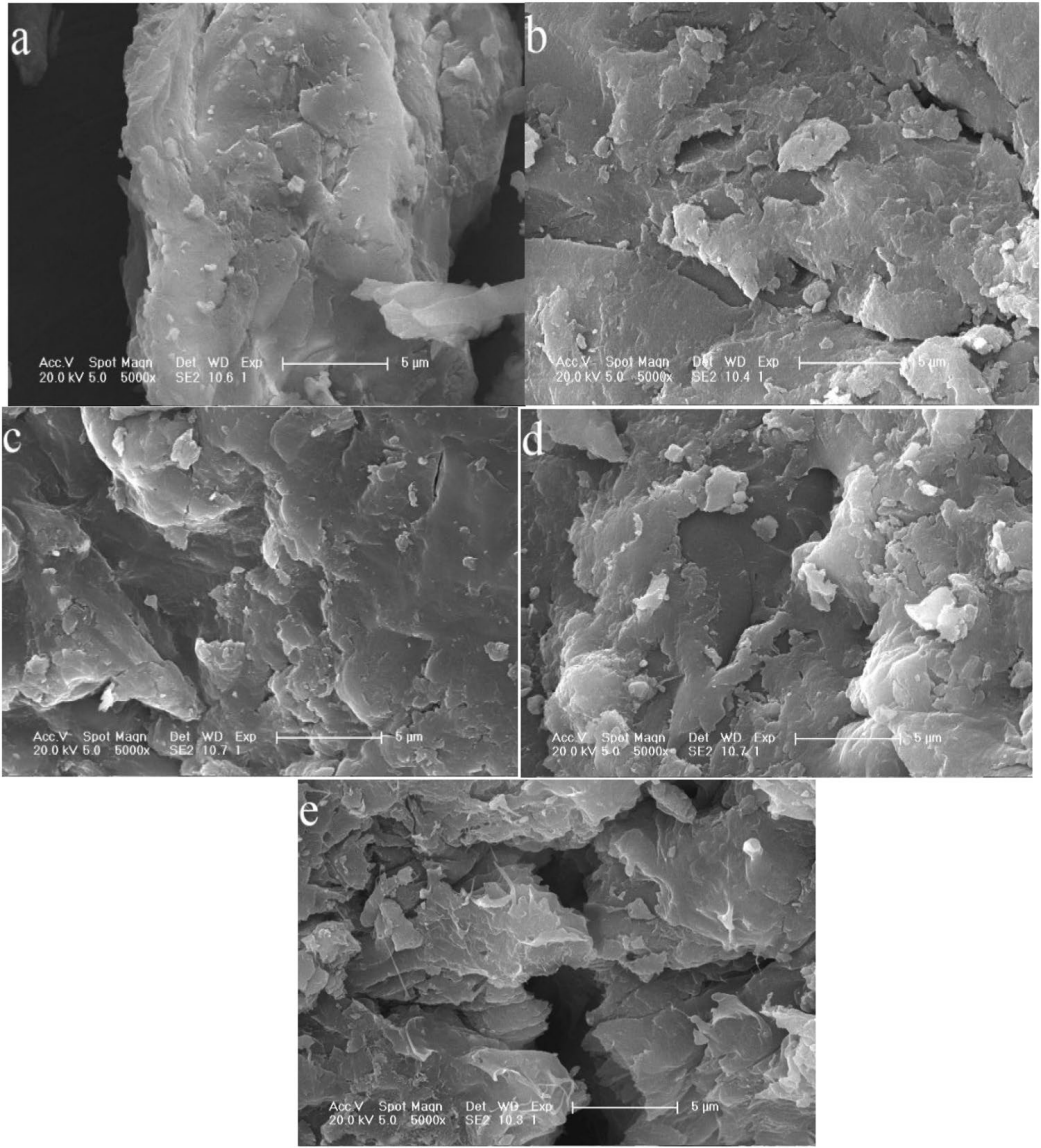
Scanning electron micrograph of degradation time of degradable soil water-retaining agent. *Note* (**a**), (**b**), (**c**), (**d**) and (**e**) are SEM photos taken before degradation, 5, 10, 15 and 30 days of degradation of degradable soil water retaining agent, respectively.

Figure 5 is the SEM results of the water retaining agent before and after degradation in soil. Figure 5a shows that the surface of the water retaining agent before degradation is smooth and flat, with complete structure. After 5 days of degradation (Fig. 5b), the surface of the water retaining agent was partially damaged, the surface structure was also rougher than that before degradation, and some holes appeared. In Fig. 5c and d, SEM images degraded for 10 and 15 days respectively, showed further damage compared with Fig. 5b. After 30 days of degradation (Fig. 5e), the surface of the water retaining agent was damaged more severely, the roughness and holes also increased significantly, and the internal structure was also damaged accordingly. The reason was that, with the gradual increase of the burial time of the water retaining agent in the soil, the microorganisms in the soil will continuously decompose the amino acids in the water retaining agent, resulting in the incomplete structure of the water retaining agent.

(4) Thermogravimetric analysis of degradable soil water retaining agent.

**Figure 6:**
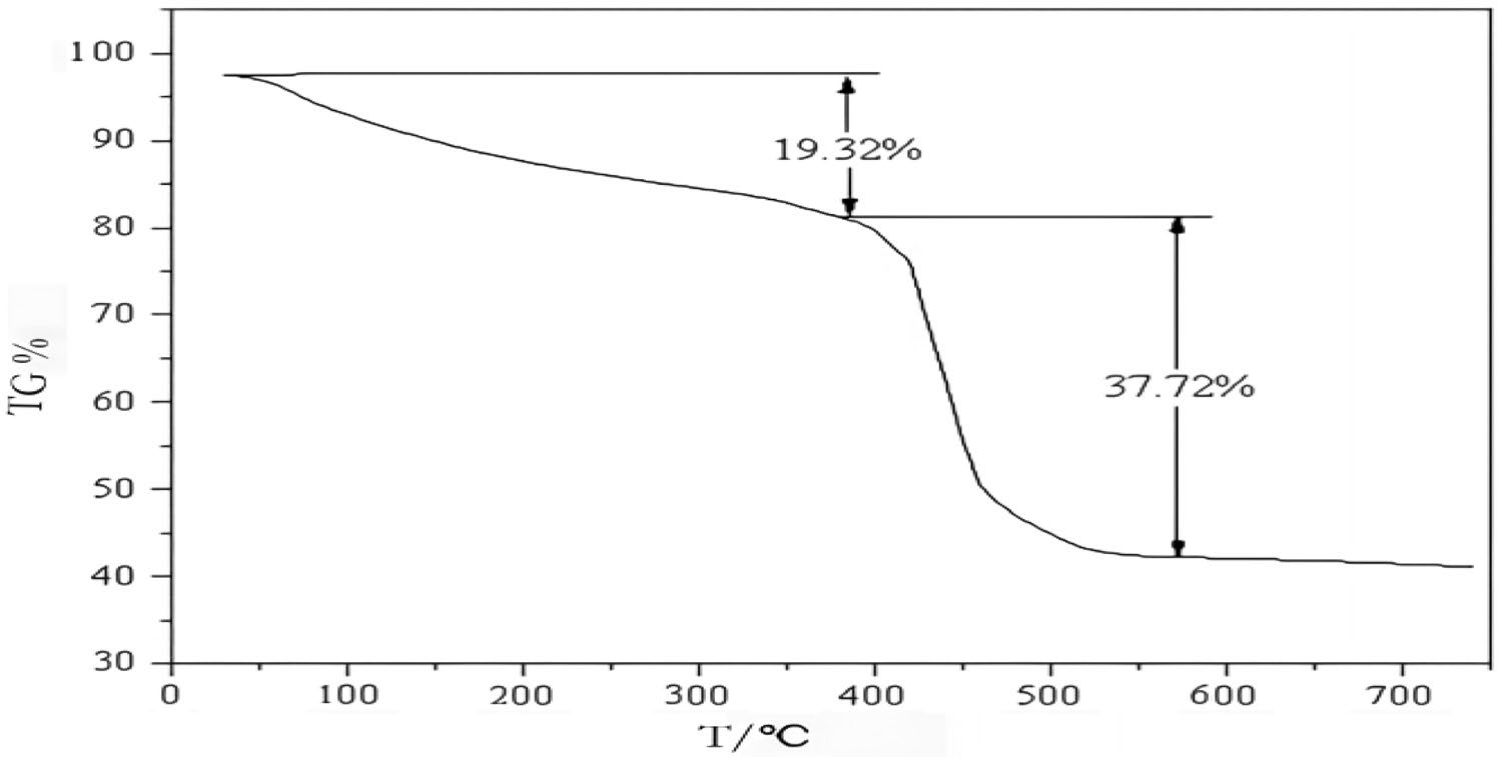
Thermogravimetric diagram of degradable soil water-retaining agent.

Figure 6 is the thermogravimetric curve of degradable soil water retaining agent. Figure 6 shows that the water retaining agent had two weight loss areas in the whole test temperature range. The first weight loss area was between 30 and 390 °C, and the weight loss rate was 19.32%, which was related to the evaporation of residual water molecules in the water retaining agent. In this weight loss range, the weight loss rate of the water retaining agent changed from fast to slow^31^. This was because the free water on the surface of the water retaining agent volatilized at the beginning, and the weight loss rate was fast, and then the bound water in the water retaining agent began to volatilize, and the weight loss rate became slow. The second weight loss zone was between 390 and 570 °C, and the weight loss rate was 37.72%. The weight loss rate was significantly accelerated, indicating that the water retaining agent started to decompose at this time, which was caused by the cross-linking network in the water retaining agent and the molecular chain breakage in the molecule. The weight of the water retaining agent at 740 °C was still 41.12% of original weight, indicating that the degradable soil water retaining agent prepared in this study had good thermal stability.

### Repetitive water absorption performance of degradable soil water retaining agent

**Figure 7:**
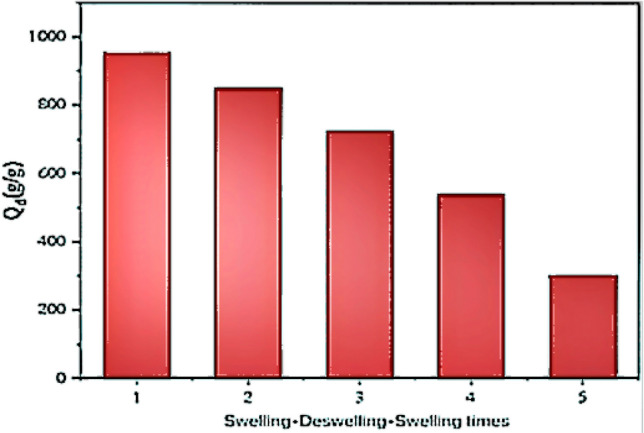
Repeated swollen of composite superabsorbent.

Studying the reabsorption performance of water retaining agent can better determine the long-term effect of water retaining agent in soil. Figure 7 shows that the first absorption rate of the water retaining agent in the deionized water was 953 g/g, and after the water was completely lost, the water retaining agent still had a strong water absorption capacity. After four times of repeated water absorption, the water absorption rate was still 302 g/g, which was about 40% of the original the water absorption rate. It indicated that the degradable soil water retaining agent had good repeated water absorption.

## Conclusions

1. In this paper, PAsp5% and BT3% were used as the main raw materials to synthesize the water retaining agent under the conditions of KPS0.3%, MBA0.03%, neutralization of 75% and microwave power of 490W. The absorbance of the synthesized water retaining agent in deionized water and 0.9% sodium chloride solution was 953 g/g and 164 g/g, respectively.
2. The synthesized water retaining agent has the characteristics of good thermal stability, degradability and good water retaining effect. It can degrade 32.75% in soil within 30 days. At the same time, it has the effect of repeated water absorption and has good water absorption rate after repeated water absorption for 4 times.
3. The water retaining agent has been applied to corn pot and achieved good results.

## Data Availability

The data used in this study is available from the corresponding author upon request.
